# Transient Receptor Potential Channels, Natriuretic Peptides, and Angiotensin Receptor-Neprilysin Inhibitors in Patients With Heart Failure

**DOI:** 10.3389/fcvm.2022.904881

**Published:** 2022-05-26

**Authors:** Kun Ding, Yang Gui, Xu Hou, Lifang Ye, Lihong Wang

**Affiliations:** ^1^Bengbu Medical College, Bengbu, China; ^2^Zhejiang Provincial People’s Hospital, Hangzhou, China

**Keywords:** transient receptor potential cation channel subfamily V member 1, transient receptor potential cation channel subfamily C member 6, natriuretic peptide, heart failure, angiotensin receptor-neprilysin inhibitor (ARNI)

## Abstract

Heart failure (HF) remains the leading cause of death, morbidity, and medical expenses worldwide. Treatments for HF with reduced ejection fraction have progressed in recent years; however, acute decompensated heart failure remains difficult to treat. The transient receptor potential (TRP) channel family plays roles in various cardiovascular diseases, responding to neurohormonal and mechanical load stimulation. Thus, TRP channels are promising targets for drug discovery, and many studies have evaluated the roles of TRP channels expressed on pain neurons. The natriuretic peptide (NP) family of proteins regulates blood volume, natriuresis, and vasodilation and can antagonize the renin-angiotensin-aldosterone system and participate in the pathogenesis of major cardiovascular diseases, such as HF, coronary atherosclerotic heart disease, and left ventricular hypertrophy. NPs are degraded by neprilysin, and the blood level of NPs has predictive value in the diagnosis and prognostic stratification of HF. In this review, we discuss the relationships between typical TRP family channels (e.g., transient receptor potential cation channel subfamily V member 1 andTRPV1, transient receptor potential cation channel subfamily C member 6) and the NP system (e.g., atrial NP, B-type NP, and C-type NP) and their respective roles in HF. We also discuss novel drugs introduced for the treatment of HF.

## Introduction

**FIGURE 1:**
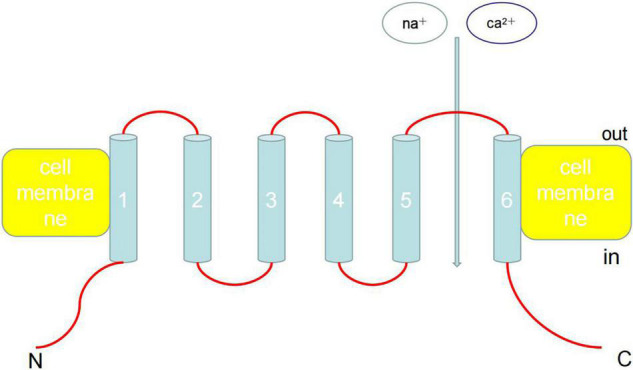
Architecture of TRP channels. TRP can be activated by various physical and chemical stimuli and consist of six transmembrane helices (TM1-6), cytoplasmic N- and C-termini, and a pore area between TM5 and TM6. Ankyrin repeats are found in the amino termini of TRPC and TRPV channels. Currently, the functions and activation mechanisms of TRP family proteins are unclear.

On October 4, 2021, American scientists David Julius and Ardem Patapoutian won the 2021 Nobel Prize in Physiology and Medicine Award owing to their outstanding contributions to the discovery of receptors that sense temperature and touch. In particular, transient receptor potential (TRP) cation channel subfamily V member 1 (TRPV1) has been shown to act as a receptor for capsaicin. TRP channel proteins can be divided into six families: canonical TRP (TRPC), vanilloid TRP (TRPV), melastatin TRP (TRPM), ankyrin TRP, mucolipin TRP, and polycystin TRP (Figure 1). These proteins can be activated by various physical and chemical stimuli and consist of six transmembrane helices (TM1–6), cytoplasmic N- and C-termini, and a pore area between TM5 and TM6 (1, 2). Currently, the functions and activation mechanisms of TRP family proteins are unclear.

The TRPV and TRPC subfamilies are the two most abundant subfamilies and include ion channels that are involved in neuronal pain pathways as well as heat sensing and permeability functions (1). TRP channels are expressed in almost all cardiovascular tissues and are a part of Ca^2+^ entry channels for store-operated channels, receptor-operated channels, stretch-activated channels, and ligand-gated channels. The functional significance of TRP channels may be related to the various gated stimuli that induce Ca^2+^ entry by integrating various variety of physical and chemical factors. Therefore, the participation of TRP channels in cardiovascular disease is uniquely relevant because it provides an opportunity to interfere with the Ca^2+^-dependent signal transduction process in the cardiovascular system. Although the roles of most TRP channels in cardiovascular diseases are largely unknown, there is no doubt that research linking TRP channel functions with cardiovascular diseases will become an important topic in the medical community in coming years (3).

Natriuretic peptides (NPs) are a group of peptides involved in maintaining the body’s water-salt balance, blood pressure, and cardiovascular and kidney function. The NP system is mainly composed of three well-characterized peptides, each of which is a different gene product with a similar structure; atrial NP (ANP) and B-type NP (BNP) are mainly derived from cardiomyocytes (4, 5), whereas C-type NP (CNP) is mainly derived from endothelial cells and kidney cells (6–9). These three peptides protect the heart and kidneys. Notably, BNP is also produced by cardiac fibroblasts and has antifibrotic effects in the heart (10). Muscle cells release ANP and BNP when the cardiac muscle is stretched, whereas endothelial cells release CNP when cytokines and endothelium-dependent agonists, such as acetylcholine, are released. Similar to ANP and BNP, CNP has powerful systemic cardiovascular effects, including reduction of cardiac filling pressure and output, secondary to reduction of vasodilation and venous return, but with minimal renal effects (11). CNPs have the strongest antifibrosis effects among the three natural endogenous NPs. All NPs operate through a second messenger, cGMP. ANP and BNP bind to guanylyl cyclase (GC)-A, whereas CNP binds to GC-B (12). Furthermore, the three NPs are all cleared by the clearance receptor NP receptor (NPR) C (NPR-C), which is unrelated to GCs (13). However, new evidence shows that NPR-C also participates in the antifibrotic effects of NP through a mechanism independent of cGMP activation (14). NPs are also eliminated from circulation by enzymatic degradation of neprilysin (NEP) (12).

Recent studies have demonstrated the roles of NPs, particularly the GC-A agonists ANP and BNP, in metabolic regulation. Among the many potentially beneficial findings, GC-A activation was shown to increase lipid oxidation in transgenic rodents, inhibit fat cell growth, increase oxygen consumption, enhance mitochondrial biogenesis in rodent skeletal muscle, delay gastric emptying, activate adiponectin, convert white adipocytes into brown adipocytes, reduce insulin levels, and improve glucose tolerance (15–23). In addition, a specific human ANP gene mutation, RS5068, increases circulating ANP levels and protects against hypertension and metabolic syndrome (24, 25). Moreover, both hypertension and obesity are related to the reduction of ANP and BNP levels, indicating that the NP system is defective in these cases; therefore, NP treatment is required (26–29). However, owing to the inherent biological properties of NPs, these hormones are now considered to be effective for the treatment of HF and other cardiovascular diseases (such as hypertension) (30–33). Accordingly, in the treatment of human cardiovascular diseases, enhancing the NP system and preventing its degradation by NEP inhibition is a goal worthy of research. The role of NEP inhibition has been explored in animal models and humans, either alone or in combination with the inhibition of other systems involved in cardiovascular disease progression.

In this review, we discuss the relationships between typical TRP family channels and the NP system as well as their respective roles in HF. We also discuss novel drugs introduced for the treatment of HF.

## Transient Receptor Potential Channel Proteins and Atrial Natriuretic Peptide

### Transient Receptor Potential Cation Channel Subfamily V Member 1 and Atrial Natriuretic Peptide

TRPV1 is a complex component of the ANP cell signaling pathway. The ANP receptor is a guanylate cyclase and antagonist of the renin-angiotensin-aldosterone system (RAAS). Interaction trap data using the amino and carboxyl termini of intracellular TRPV1 as bait suggested that TRPV1 interacts with NPR1 (GC-A) (13, 34). Activation of the NPR1 and ANP pathway leads to cGMP-dependent stimulation of TRPV1 phosphorylation, thereby inhibiting TRPV1 and reducing the surface current. NPR1-knockout experiments effectively demonstrated the presence of the ANP/NPR1 antihypertrophic pathway in the heart (35). Therefore, the ANP/NPR1/cGMP/protein kinase G (PKG)/TRPV1 linkage mechanism may represent a target for the antifertilization effects of ANP/NPR1. However, further studies are needed to clarify the effects of PKG phosphorylation on TRPV1 (sGC-PKG) because there are two ways to regulate cardiomyocyte function. The PKG pathway, which is regulated by sGC (NO) and pGC (NP), involves PKG phosphorylation, and PKG negatively regulates TRPV1. Data from our recent studies support the negative regulatory effects of NPR1/PKG/TRPV1 phosphorylation; however, in general, compared with protein kinase A and protein kinase C (PKC), the effect of PKG phosphorylation on TRPV1 have not been extensively studied (36–38).

### Transient Receptor Potential C6 and Atrial Natriuretic Peptide/B-Type Natriuretic Peptide

In our recent studies, we used an *in vitro* culture system and an *in vivo* genetic engineering model to reveal the functional negative correlations between the ANP/GC-A/cGMP/PKG and TRPC6 calcineurin/fat pathways in cardiomyocytes. ANP directly inhibits the activity of TRPC6 through the cGMP/PKG pathway, thereby blocking the hypertrophic signaling pathway. The selective TRPC inhibitor BTP2 significantly reduces myocardial hypertrophy in GC-A-knockout mice, which are sensitive to the hypertrophic signals caused by TRPC6 overexpression. BTP2 significantly inhibits myocardial hypertrophy caused by chronic infusion of angiotensin II (Ang-II). PKG also inhibits L-type calcium channel (LTCC) activity and calcineurin/nuclear factor of activated T cells (NFAT) signaling (39). In fact, activation of TRPC3/6 has been shown to lead to the activation of LTCC (40), and ANP may inhibit this activation, thereby suppressing calcineurin/NFAT signal transmission. Therefore, the ANP/BNP/cGMP/PKG signaling pathway may inhibit the hypertrophic signaling pathway through multiple steps. ANP and BNP have been used clinically in patients with acute heart failure (31, 41), and the ANP/GC-A/cGMP/PKG signal transduction pathway is a complex pathway involving multiple mechanisms. Therefore, our research shows that inhibition of TRPC6 activity mediates the antihypertrophic effects of ANP/BNP and suggests that inhibition of TRPC6 may prevent pathological effects, providing insights into potential effective treatment strategies for myocardial hypertrophy and remodeling (42).

## Transient Receptor Potential Channel Proteins and B-Type Natriuretic Peptide

Among membrane proteins that detect harmful stimuli, sensory neurons (pain receptors) of small and medium diameters express capsaicin (and heat)-sensitive TRP and allicin-1 (TRPV1) channels and/or ATP-gated P2 × 3 subunit receptors (43, 44) to transmit pain. Some studies have shown that TRPV1 is essential for the development of inflammatory heat pain (45–47). The activity of TRPV1 and P2 × 3 receptors is upregulated by endogenous peptides, such as bradykinin, calcitonin gene-related peptide, substance P, and nutritional factors (48–52). The functional effects of these modulators manifest as receptor sensitization, thereby helping to lower the pain threshold and trigger pain, particularly chronic pain.

The NP family, including ANP, BNP, and CNP, are involved in pain sensing. ANP does not affect sensitivity to radiant heat (53) or mechanical ectopic pain (54, 55), whereas CNP is considered an active regulator of chronic pain (56). By contrast, microarray genetic analysis have shown that chronic pain enhances BNP expression and NPR-A, a receptor for BNP, in the rat dorsal root ganglion (DRG). In addition, in a rat model of inflammatory pain, application of BNP reduced the excitability and hyperalgesia of DRG allodynia receptors, suggesting that BNP may have inhibitory roles in chronic pain (57). BNP functions by binding to NPR-A, which is a guanosine cyclase receptor (also sensitive to ANP), and increases intracellular cGMP levels (58, 59). These studies have not explored the molecular mechanisms of GC-A activation and TRPV1 and P2 × 3 receptor changes, and further research is therefore needed to demonstrate whether and how BNP regulates TRPV1 and P2 × 3.

Several intracellular cascades are involved in the trigeminal ganglion (TG). Moreover, TG neurons strongly express NPR-A (in the absence of BNP binding), making TG particularly sensitive to BNP released from various peripheral tissues under physiological and pathological conditions (60). Therefore, the NPR-A system may act as a slow regulator of sensory excitability.

## Transient Receptor Potential Channel Proteins and C-Type Natriuretic Peptide

NPR is an enzyme-linked receptor (GC); NPR-A and NPR-B mediate the conduction effects of all NPs in *via* a conventional GC/cGMP/PKG pathway. However, unlike NPR-A and NPR-B, NPR-C lacks the GC kinase domain and therefore acts as an NP clearance receptor (61, 62). Our research shows that functional NPR-A/B/C is expressed in mouse DRG neurons, and CNP and ANP, but not BNP, induce sensitization of TRPV1 channel activity, independent of classic NPR-A/NPR-B/cGMP/PKG signaling. By contrast, NP sensitization of TRPV1 proceeds through atypical NPR-C/Gβγ/phospholipase C (PLC) β/PLC-mediated signaling modules. Specific drugs block these signaling components, and CNP attenuates the regulation of TRPV1 activity. This modulation of TRPV1 channel activity causes nociceptors to be sensitive to mildly and pathophysiologically related acidic conditions. However, this modulatory mechanism is absent in TRPV1-deficient DRG neurons. In addition, plantar injection of CNP induces thermal hyperalgesia in wild-type mice, but not in TRPV1-deficient mice. Gβγ and TRPV1 inhibitors can be systemically administered to modulate these effects. Overall, these findings indicate that CNP is involved in a novel non-classical Gβγ/PLCβ/PKC/TRPV1 signaling pathway to induce hyperalgesia and thermal hyperalgesia, which may occur under various tissue damage and inflammatory conditions (56).

In general, NPs have substantial effects on inflammatory heat and mechanical allergies. The NPR-A/NPR-B/cGMP/PKG signaling pathway in the central axon of DRG neurons projected to the dorsal horn of the spinal cord promotes mechanical ectopic pain (55, 63, 64), whereas intrathecal BNP induces thermal hyperalgesia and has analgesic effects (57). Our research shows that the PKC/TRPV1 signal in the peripheral afferent nerve is involved in the occurrence of thermal hyperalgesia *via* CNP/Gβγ and NPR-B/NPR-C, which is widely expressed in neurons of all sizes in the DRG. Additionally, the increase in cGMP production in CNP-induced neurons jointly indicates that CNP may be involved in the peripheral sprouting/bifurcation of sensory afferent nerves and induces mechanical effects, including dyskinesia and hyperalgesia. Further studies are required to verify these findings (56).

CNP and ANP have higher affinities for NPR-C than BNP (14, 65). This can explain the lack of BNP-induced TRPV1 currents observed in our work. Based on these findings, we propose a signal module mediated by NPR-C/Gβγ/PLCβ/PLC (56). High concentrations of morphine can directly activate and enhance the TRPV1 currents in mouse DRG neurons (66). Considering that many opioid receptors induce Gαi-coupling downstream signals (67, 68), the sensitization of TRPV1 channel activity by high doses of morphine (66) may involve the Gαi/Gβγ/PLCβ/PKC signaling pathway. This hypothesis still needs to be verified experimentally.

## Transient Receptor Potential Channel Proteins and Heart Failure

Calcium ions participate in many physiological reactions in the human body and play important roles in many cellular reactions. For example, calcium acts as second messenger and blood coagulation factor IV, participates in excitation and contraction coupling, and modulates muscle contraction. Altered intracellular Ca^2+^ helps modulate impaired systolic HF (69, 70). In the myocardium, intracellular Ca^2+^ storage and Ca^2+^ ATP enzymes [sarco endoplasmic reticulum Ca^2+^-ATPase (SERCA) 2 subtype] play important roles in contraction activation and relaxation. However, in HF, an increase in sodium-calcium exchange (NCX) levels is associated with downregulation of SERCA. In addition, an *in vitro* study using small interfering RNA against myocardial SERCA showed that decreased SERCA2 expression is related to TRPC4, TRPC5, and NCX upregulation, suggesting that deficiency of intracellular storage may be compensated for by entry of Ca^2+^ through the plasma membrane (71). In fact, induced expression of TRPC5 or TRPC6 can be observed in patients with HF (72, 73). The importance of this compensatory mechanism may be related to its universal participation in the Ca^2+^ signaling mechanism, not only for excitation-contraction coupling but also for heart remodeling and hypertrophy.

Myocardial apoptosis is an important process leading to HF; therefore, inhibition of apoptosis is a promising treatment. Apoptosis is induced by various stimuli, including oxidative stress, pro-inflammatory cytokines, catecholamines, and Ang-II (74). Elevated intracellular Ca^2+^ is considered a key initiator of intracellular apoptosis signal transduction (for example, activation of endogenous endonucleases dependent on Ca^2+^). Currently, there are two reports of TRP channels being involved in myocardial apoptosis in animal models. Activation of the TRPM2 channel and poly (ADP-ribose) polymerase (PARP) is involved in cardiomyocyte death induced by oxidative stress (75, 76). The apoptotic component is caused by activation of the clotrimazle-sensitive NAD^+^/ADP ribose/PARP-dependent TRPM2 channel, which induces mitochondrial Na^+^ and Ca^2+^ overload, leading to mitochondrial membrane rupture, cytochrome release, and caspase-3-dependent chromatin condensation/fragmentation. Moreover, TRPC7 may be the key initiator of AT1 activation, leading to myocardial apoptosis, and could then participate in the progression of HF (77). Although these findings provide possible strategies for regulating cardiac apoptosis, further research is needed to clarify the complex relationships between TRP and HF.

## Natriuretic Peptides and Heart Failure

NPs, particularly BNP, play critical roles in the diagnosis of HF. The accuracy of HF severity assessment is directly related to clinical outcomes of treatment in patients. BNP levels are the most commonly used index in clinical practice. The diagnostic roles of NPs in acute and chronic HF have been confirmed. In particular, ANP and BNP levels increase in parallel with the degree of left ventricular dysfunction and hemodynamic pressure, although they are not helpful in distinguishing between systolic and diastolic HF (78). In addition, in cases of acute and chronic HF, the value of NPs as reliable markers for long-term prognostic stratification has been demonstrated. In a study of patients with acute HF, BNP, NT-proBNP, and mid-region (MR) proBNP levels showed significant diagnostic performance, although only MR-proBNP had long-term prognostic value (79). In patients hospitalized due to acute decompensated HF (ADHF), the prognostic performance of NT-proBNP and MR-pro-ANP levels was confirmed by evidence of the increased prognostic value of clinical risk factors for predicting mortality within 1 year of onset (80). In patients with chronic HF, subsequent measurements of BNP or NT-proBNP levels provide independent information about the risk of disease progression, which involves a series of adverse consequences, including ventricular remodeling, malignant ventricular arrhythmia, HF-related hospitalization, transplantation need, and death (81). In the longest follow-up study of patients with chronic HF, the prognostic ability of multiple biomarkers was evaluated, and the results showed that the plasma ANP level was the strongest long-term predictor of death during all disease stages (82). NPs have prognostic value in patients with HF with reduced ejection fraction (HFrEF) and HF with retention of ejection fraction (HFpEF) (83). Recent studies have shown that screening based on BNP has preventive effects on HF development. In patients with cardiovascular risk factors and those at risk of HF, BNP assessment related to combination therapy reduces the combined incidence of left ventricular systolic dysfunction, diastolic dysfunction, and HF (84).

The clinical significance of NPs in HF indicates that NP level measurement is an effective tools for hormone-guided treatment of HF. In ADHF, BNP measurement improves the accuracy of diagnosis, reduces the hospitalization and admission rates in the intensive care unit, and has beneficial effects on treatment costs and mortality (85). A useful algorithm was developed to treat ADHF guided by BNP (85). Additionally, in chronic HF, NP levels are useful markers for monitoring disease progression related to the benefits of different treatment strategies (86). However, because of uncertainty among studies, the American Heart Association/American College of Cardiology HF guidelines recommend a low level of NP-guided treatment for patients with chronic HF (87). A recent meta-analysis attempted to overcome existing controversies, including randomized clinical trials, which showed that in patients with chronic HF, NP-guided treatment reduces all-cause mortality and HF-related hospitalization (88).

## Angiotensin Receptor-Neprilysin Inhibitors and Heart Failure

**FIGURE 2:**
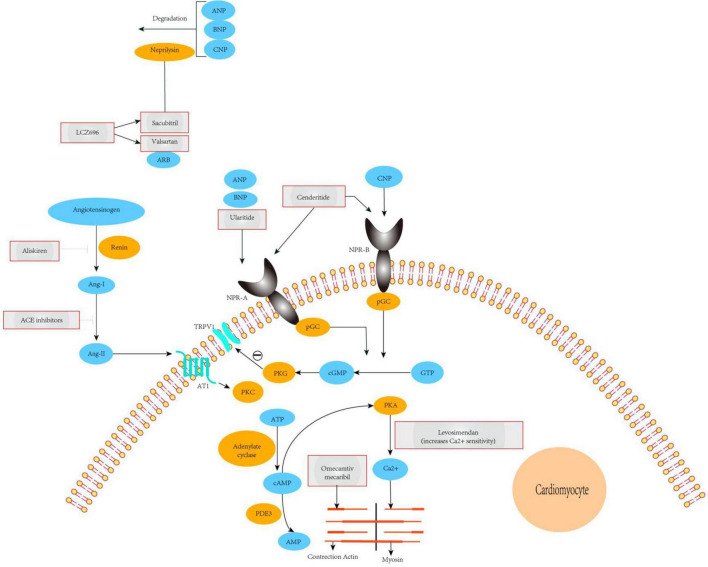
Mechanisms of action of novel therapeutics for heart failure. The ARNi LCZ696 is split into the ARB valsartan and the neprilysin-inhibitor sacubitril. Valsartan abrogates signaling *via* the AT1 receptor, inhibiting deleterious effects mediated by Ang-II such as vasoconstriction, hypertrophy, and fibrosis in major cardiovascular organs. Sacubitril prevents breakdown of endogenous natriuretic peptides (ANP, BNP, and CNP), thereby augmenting their beneficial actions in cardiovascular disease. The overall effects of ARNi are vasodilatation, natriuresis, and diuresis, as well as inhibition of fibrosis and hypertrophy. Ularitide selectively targets NPR-A, whereas cenderitide activates both NPR-A and NPR-B. Both peptides increase intracellular cGMP, which in turn leads to inhibition of the renin-angiotensin-aldosterone system and attenuation of fibrosis, hypertrophy, and vasoconstriction. Our proposed model shows that TRPV1 interacting with NPR-A, activation of the NPR1 and ANP pathway leads to cGMP-dependent stimulation ofTRPV1 phosphorylation. PDE3 antagonists (enoximone, milrinone), calcium sensitizers (levosimendan), and a direct activator of cardiac myosin (omecamtiv mecarbil). ACE, angiotensin-converting enzyme; TRPV1, transient receptor potential cation channel subfamily V member 1; Ang-I/II, angiotensin I/II; ARB, angiotensin-receptor blocker; ARNi, angiotensin receptor-neprilysin inhibitor; AT1, type-1 angiotensin II receptor; ANP, A-type natriuretic peptide; BNP, B-type natriuretic peptide; CNP, C-type natriuretic peptide; NPR-A/B, atrial natriuretic peptide receptor 1/2; PDE3, phosphodiesterase-3; pGC, plasma- membrane-bound guanylate cyclase; PKA, protein kinase A; PKC, protein kinase C; PKG, protein kinase G; pGC, plasma- membrane-bound guanylate cyclase; sGC, soluble guanylate cyclase.

NEP is a neutral endopeptidase responsible for the degradation of endogenous vasoactive substances in the body, including NPs (ANP, BNP, and CNP), bradykinin, and adrenal medulla hormones. BNP, which is synthesized and secreted by ventricular myocytes, can promote sodium excretion and diuresis and has strong vasodilator effects. The use of pharmacological drugs to reduce the catabolism and elimination of endogenous NPs has been applied in clinical practice. Indeed, sacubitril combined with valsartan to inhibit NEP has been shown to be a successful method for the treatment of arterial hypertension, chronic congestive HF, and myocardial fibrosis. Sacubitril and valsartan sodium are novel types of anti-HF drugs. Valsartan is an angiotensin receptor antagonist that inhibits the RAAS, thereby expanding blood vessels, lowering blood pressure, inhibiting myocardial remodeling, and blocking abnormal activation of the nerve and endocrine system. By contrast, sacubitril is an NEP. The three types of NPs are inactivated by receptor-mediated internalization and lysosomal degradation, followed by enzymatic neutral endopeptidase or NEP degradation. Recent studies have shown that, compared with enalapril, NEP inhibition can moderately increase BNP levels and enhance levels of endogenous brain NP, thereby dilating blood vessels, inducing diuresis, improving myocardial remodeling, and exerting other cardioprotective effects. The combination of the two constitutes sacubitril and valsartan sodium can be used for the treatment of various cardiovascular diseases, including hypertension, HF, and coronary heart disease (Figure 2). Sacubitril and valsartan sodium can also inhibit the adverse effects of the RAAS and activate the cardiovascular protective effect of enkephalins. Therefore, drugs involving the combination of both of these components may be suitable for the treatment of patients with HF.

In a prospective study, the effects of angiotensin receptor-neprilysin inhibitors and angiotensin-converting-enzyme inhibitors (ACEIs) on the global mortality and morbidity of heart failure (PARADIGM-HF) were compared. In this multicenter trial, 8,442 patients with reduced ejection fraction and NYHA class II–IV were randomly assigned to the treatment group and received valsartan/sacubitril 200 mg/2 days or enalapril 10 mg/2 days (89–91). After a median follow-up of 27 months, 21.8% of patients in the valsartan/sacubitril group died from cardiovascular causes or were hospitalized for HF compared with 26.5% in the enalapril group; the risk was reduced by 20% (*p* < 0.001) (89). There were no differences between the two groups in the incidence of new-onset atrial fibrillation or decreased renal function. In terms of drug side effects, the incidence of symptomatic hypotension in the valsartan/sacubitril group was higher than that in the enalapril group (14% versus 9.2%, *p* < 0.001), whereas patients in the enalapril treatment group had cough and increased serum creatinine (2.5 mg/dL or higher). Additionally, the incidence of hyperkalemia was significantly higher in the valsartan/sacubitril group than in the enalapril group. During the double-blind treatment, 19 patients in the valsartan/sacubitril group developed angioedema, and 10 patients in the enalapril group developed angioedema. Compared with ACEIs alone, the combined use of ACEIs and the heparanase inhibitor omapatrilat increased the risk of angioedema threefold (92). Thus, valsartan/sacubitril, the first combined ARNI/nephrase inhibitor, can reduce mortality in patients with HFrEF. Ongoing clinical trials will determine its efficacy in other conditions, including hypertension and HFpEF (93).

At present, heart failure is divided into three categories. The first category is heart failure with preserved ejection fraction, the second category is heart failure with reduced ejection fraction, and the third category is heart failure with intermediate ejection fraction. The diagnosis standard needs to be calculated according to 50 and 40%, that is, if the patient’s ejection fraction ≥50%, it belongs to heart failure with preserved ejection fraction. If the patient’s ejection fraction is less than or equal to 40%, it is classified as heart failure with reduced ejection fraction. If the patient’s ejection fraction is between 40 and 50%, 50 and 40% are not included, which belong to the heart failure in the middle position of the ejection fraction. Under normal circumstances, the earliest occurrence of heart failure is diastolic dysfunction, and with the gradual decline of diastolic function, it will gradually progress to heart failure with median ejection fraction or heart failure with reduced ejection fraction. Heart failure with preserved ejection fraction refers more to the early stage of heart failure.

For patients with chronic HFrEF, ACEI, ARB, and ARNI can be used as first-line therapy. The PARADIGM-HF study confirmed that ARNI can bring greater benefit to patients with chronic HFrEF than ACEI; but a large number of previous studies have confirmed the efficacy of RAAS inhibitors in patients with HFrEF, but these two types of drugs have poor efficacy in patients with HFpEF, which may be due to the low importance of RAAS system abnormalities in the pathophysiological mechanism of HFpEF. Among them, ARNI can significantly reduce the rate of heart failure hospitalization, but fails to reduce the composite endpoint of cardiovascular death and total heart failure hospitalization in patients with heart failure with LVEF ≥ 45% (94).

## Summary and Perspectives

The TRP family is closely related to the NP family; however, the specific molecular mechanisms remain unclear. TRPs are activated by similar prohypertrophic or profibrotic stimuli and are related to or interact to activate hypertrophy-, fibrosis-, and conduction disorder-related signaling pathways. Unfortunately, common agonists and antagonists used to modulate TRP have not been able to determine which TRP channel proteins may be the correct targets and potential therapeutic tools. Decades of research have outlined the important contributions of NPs in cardiovascular diseases. In addition to evaluating their diagnostic functions, particularly in HF, TRP channel proteins have important predictive significance for HF and hypertension. Based on the extensive effects of these hormones in the cardiovascular system, NP-derived treatments are currently considered reasonable treatments for cardiovascular diseases. Drugs to treat HF have always been limited and are typically based on RAAS and SNS suppression. In recent years, with the development of ARNI, i.e., sacubitril/valsartan (LCZ696), as the first combined angiotensin receptor antagonist/renal protease inhibitor, the mortality rates in patients with HFrEF have decreased, and its effectiveness and safety have been confirmed. However, the specific mechanisms are still unclear. Moreover, it is still unknown whether the clinical benefit of valsartan/sacubitril is driven by reducing the degradation of NPs or whether other mechanisms function to alleviate symptoms of HF by combining angiotensin receptor antagonists and renin inhibitors. In addition, ARNI is used in patients with clinical HF, and the incidence of angioedema during treatment is still not known. Further studies are needed to address these questions and improve our understanding of clinical HF in order to establish novel treatment strategies in these patients.

## Author Contributions

KD contributed to ideas, formulation, and evolution of the research goals. XH imagined and made the figures. YG and LY contributed to the preparation of the published work and especially writing the initial part. LW ensured that the descriptions are accurate and agreed by all authors.

## Conflict of Interest

The authors declare that the research was conducted in the absence of any commercial or financial relationships that could be construed as a potential conflict of interest.

## Publisher’s Note

All claims expressed in this article are solely those of the authors and do not necessarily represent those of their affiliated organizations, or those of the publisher, the editors and the reviewers. Any product that may be evaluated in this article, or claim that may be made by its manufacturer, is not guaranteed or endorsed by the publisher.
